# Quantitative evaluation of swallowing function in Parkinson’s disease using tongue pressure measurement: a mini-review

**DOI:** 10.3389/fneur.2024.1355627

**Published:** 2024-02-13

**Authors:** Tatsuyuki Fukuoka, Kazuhiro Hori, Takahiro Ono

**Affiliations:** ^1^Department of Rehabilitation, Faculty of Rehabilitation, Hiroshima International University, Hiroshima, Japan; ^2^Division of Comprehensive Prosthodontics, Faculty of Dentistry and Graduate School of Medical and Dental Sciences, Niigata University, Niigata, Japan; ^3^Department of Geriatric Dentistry, Osaka Dental University, Osaka, Japan

**Keywords:** Parkinson’s disease, dysphagia, tongue pressure, tongue strength, swallowing

## Abstract

Dysphagia is a common symptom of Parkinson’s disease (PD) associated with aspiration pneumonia, choking, malnutrition, and a decreased quality of life, and is a leading cause of death among patients with PD. Tongue dysfunction in patients with PD affects the oral phase of swallowing, including the formation and propulsion of a bolus into the pharynx. Assessing tongue pressure, generated between the tongue and palate, is a method that quantitatively measures tongue function and is related to dysphagia in PD. Two assessment methods are used to measure tongue pressure: tongue strength and tongue pressure during swallowing. Previous studies measuring tongue pressure in PD have reported decreased tongue strength and pressure during swallowing, as well as a prolonged tongue pressure rise time, which are symptoms associated with PD severity and dysphagia. In this mini-review, we present a method for measuring tongue pressure and discuss its relationship with dysphagia in PD. We also describe limitations and future perspectives in tongue pressure measurement research.

## 1 Introduction

Dysphagia occurs frequently in Parkinson’s disease (PD), not only leading to a decline in the quality of life related to meals, depression, and malnutrition, but also serving as a cause of aspiration pneumonia, which is associated with life prognosis (1–6). In PD, bradykinesia, hypokinesia, and tremors can also influence swallowing organ motility and cause problems in the oral phase, in which a high prevalence of abnormal tongue movement is typically observed (7, 8). Tongue tremors, pumping-like movements, prolonged tongue elevation, and muscle weakness are also observed, and these motor abnormalities can cause difficulty initiating swallowing, prolonged oral transit time, difficulty propelling a bolus from the oral cavity to the pharynx, a decreased propulsive force, and oral residuals (9, 10). Given these considerations, it is important to establish a clinical marker of abnormal tongue movement in the evaluation of dysphagia in PD.

The tongue plays an important role in propelling a food bolus from the oral cavity to the pharynx during swallowing, and the decreased production of tongue pressure is a risk factor for impaired safety and efficiency during swallowing (11). Tongue pressure, a measure of the pressure produced between the tongue and palate, is an indicator of tongue motility. In patients with PD, abnormal tongue pressure patterns and movements during swallowing have been reported. Measuring tongue pressure is a less invasive assessment method and may have diagnostic value in the evaluation of dysphagia in patients with PD. Given this background, the present mini-review aimed to summarize the research literature on the measurement of tongue pressure in patients with PD. A literature search was performed on PubMed, Web of Science, and Google Scholar using the terms “Parkinson’s disease” AND “tongue pressure” OR “lingual pressure” OR “tongue strength.” We reviewed the title and abstract of each result and selected articles related to tongue pressure studies in patients with PD.

## 2 Types of methods for measuring tongue pressure

There are two types of tongue pressure: maximal tongue pressure, which occurs when the tongue is voluntarily pushed strongly upward, and tongue pressure during swallowing, which is produced between the tongue and palate during swallowing. Researchers have used various terms to refer to maximal tongue pressure, including tongue strength, lingual strength, maximal lingual pressure, maximal tongue pressure, and tongue pressure strength (12–15). Because both types of tongue pressure are performed using maximal isometric movement, they are used to indicate tongue strength. In this paper, to avoid confusion among readers, the term tongue strength is used throughout.

Tongue pressure during swallowing is a method of measuring the pressure, location, and timing of tongue contact with the palate by a sensor placed in the intraoral cavity during the swallowing of saliva, food, or drinks.

## 3 Measurement of tongue strength

### 3.1 Measurement instruments and methods

**Figure 1:**
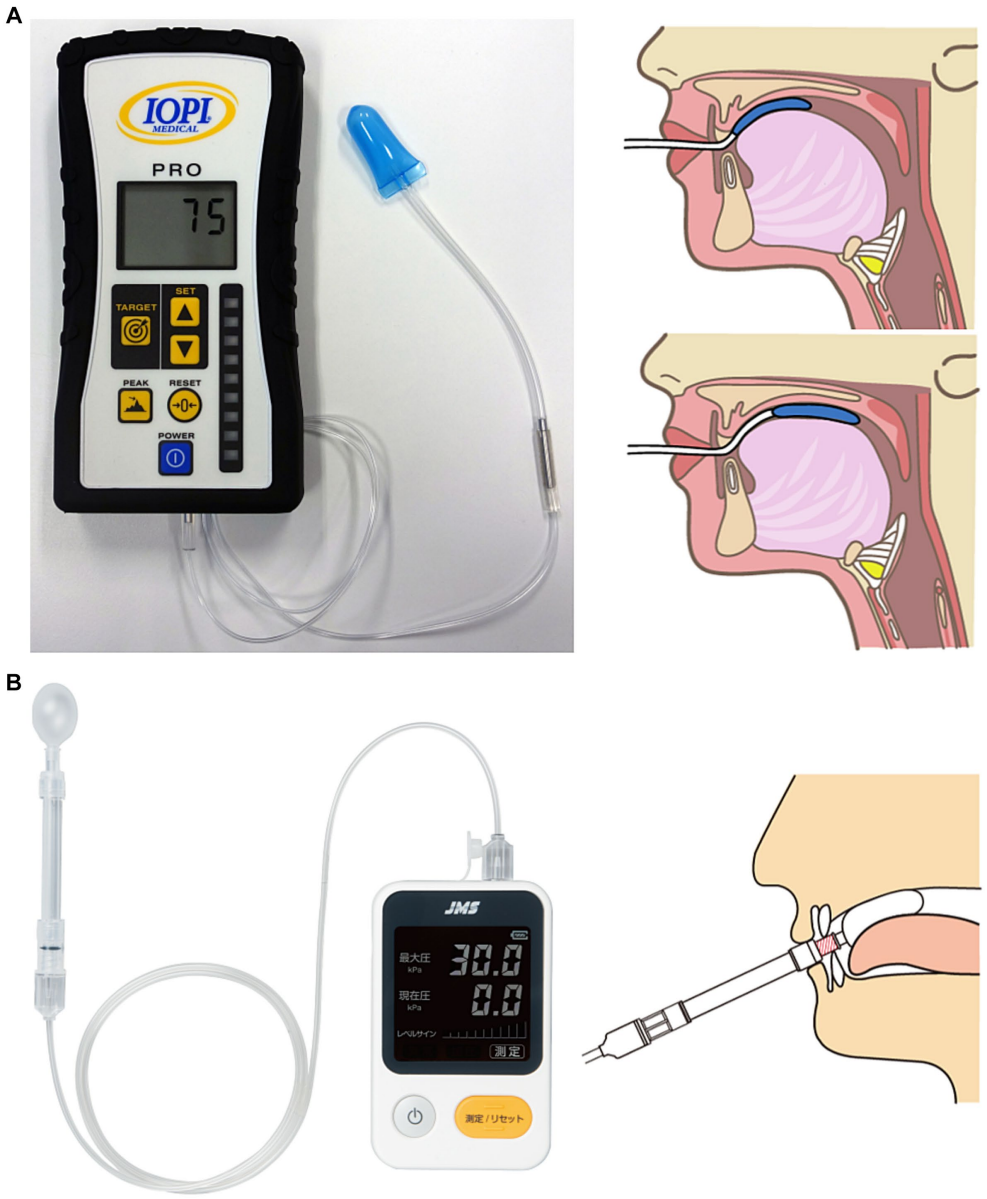
**(A)** IOPI Pro (Model 3.1) and measurement methods. **(B)** JMS Tongue Pressure Measurement Device (TPM-02) and measurement methods.

Tongue strength is measured using a tongue pressure measuring device that consists of an instrument unit, a connecting tube, and a tongue pressure probe. A probe with a balloon-shaped tip is placed on the tongue in the oral cavity and pressed with maximal force against the palate to measure the pressure produced. The subject is instructed to “press as hard as you can with your tongue against the plastic bulb.” Measurements are taken three times and the average or maximal value is recorded as the representative value for each subject. A typical tongue pressure measuring device is the IOPI Pro (Model 3.1; IOPI Medical LLC, Woodinville, WA, United States), which is the most widely used device and has been the subject of many research reports (16–19) (Figure 1A). The IOPI is a handheld portable device that uses an air-filled plastic bulb (3.5 cm long; 4.5 cm diameter, 2.8 mL internal volume) (20). Another common device is the JMS Tongue Pressure Measurement Device (TPM-02; JMS Co., Hiroshima, Japan), which is used mainly for clinical and research purposes in Japan (21–25) (Figure 1B). The tip of the IOPI probe has a slightly flattened shape, whereas that of the JMS device has a nearly spherical shape. The JMS device has a rigid ring at the base of the balloon that is secured by the incisors to control mandibular movement and balloon positioning. Because the IOPI probe has no fixed points on the incisors, the probe position can be moved from the anterior to posterior portion of the tongue. Both the IOPI and JMS instruments are expressed in kilopascals (kPa), but because the instruments are different, the readings are not directly comparable. A conversion formula for the relationship between the tongue strength value of both devices has been reported by Yoshikawa et al. (26). Real-time and tongue strength value displays are common features of both devices.

These instruments can also assess endurance by measuring not only tongue strength, but also the time it takes to keep the tongue pressed with a constant force. Tongue endurance is measured by the time that 50% of maximal tongue strength can be sustained with feedback of the pressure ramp displayed on the tongue pressure measuring device and the waveform displayed on the monitor using specialized software (27, 28).

These tongue pressure measuring devices can also be used for tongue strength training, and have been applied to rehabilitation therapy using tongue strength values as an indicator.

### 3.2 Tongue strength in PD

A number of studies have shown that patients with PD have decreased tongue strength and endurance compared with controls in similar age groups (29–31). Solomon et al. (30) investigated muscle strength and endurance in the tongue and hands of patients with PD and compared them with neurologically normal controls, and reported that the patients with PD had 8.3 kPa lower tongue strength and 8.2 s shorter endurance. Tongue strength can be measured in the anterior and posterior portions of the tongue, but studies of PD have reported declines in only the anterior portion or in both the anterior and posterior portions (32, 33).

Regarding the relationship with the progression of PD, tongue strength has been shown to decrease more in patients with advanced than in patients with mild/moderate disease progression. Plaza et al. (34) reported a negative correlation between Hoehn and Yahr stage and tongue strength, which decreases with the progression of PD. They reported finding no differences in tongue endurance or gender based on the degree of PD progression. According to a meta-analysis by Pitts et al. (31), decreased muscle strength and endurance in the anterior part of the tongue is expected in approximately one-third and one-fourth of patients with PD, respectively. They point out that the decrease in the anterior part of the tongue appears from stage II of the Hoehn and Yahr classification and may become more persistent as the disease progresses.

An analysis of the relationship between tongue pressure and swallowing function using videofluoroscopic swallowing studies reported that higher tongue strength leads to less airway penetration of thin liquids, and that low endurance in the anterior portion of the tongue delays the laryngeal vestibule closure time (33, 35).

Weak tongue strength is also associated with subjective symptoms of dysphagia. Tongue strength is decreased in patients with PD with subjective symptoms of dysphagia compared with those without PD (36). Pitts et al. (32) reported that patients with PD with reduced muscle strength in the anterior part of the tongue had lower total scores on the Swallowing Quality of Life (SWAL-QOL) questionnaire and reported subjective symptoms such as prolonged eating time and decreased motivation to eat.

De Letter et al. (37) examined the effect of levodopa on tongue pressure. Ten patients with idiopathic PD were studied for isometric motor tasks of the tongue in the on and off phases. The maximal force of tongue movement and contraction time were not significantly different between the two conditions, but the integral (area under the curve) was significantly greater in the on phase. In addition, the force decay slope was significantly lower in the on phase. These findings are consistent with the pathophysiological effects of isometric muscle contraction patterns in PD.

## 4 Measurement of tongue pressure during swallowing

### 4.1 Measurement instruments and methods

Tongue pressure during swallowing is a method of measuring the contact pressure between the tongue and palate during swallowing. A simple method is to place the balloon of a tongue-pressure measuring device such as the IOPI on the tongue and measure the pressure of the tongue pushing the balloon upward during swallowing (33, 35). A detailed evaluation of tongue pressure during swallowing involves attaching a customized sensor to the palate and measuring the contact between the tongue and the palate. Multiple pressure sensors are installed in the palate, including in the bulb and mouthpiece (38–40). The tongue pressure sensor (Nitta Co., Osaka, Japan) has five pressure-sensitive sensors arranged in an ultra-thin sheet (0.1 mm thick), allowing it to measure tongue pressure during swallowing under natural conditions with minimal discomfort (39, 40). The measurement of tongue pressure during swallowing provides detailed objective information on the site of tongue contact with the palate, the order of tongue pressure onset, and the maximal tongue pressure, duration, and integral value for each site.

### 4.2 Tongue pressure during swallowing in PD

Studies using IOPI bulbs have shown that tongue pressure during salivary swallowing is decreased in patients with PD compared with healthy older adults (11, 35). Da Costa et al. (33) compared tongue pressure during salivary swallowing in 23 patients with idiopathic PD (mean age, 64.9 years) and 24 healthy controls (mean age, 64.1 years) using the IOPI placed anteriorly and posteriorly. They reported that compared with the control group, patients with PD had significantly lower tongue pressure during swallowing in the posterior region.

Hadley et al. (38) measured real-time tongue-palatal pressure in patients with PD using an oral mouthpiece with seven implanted pressure sensors. Using this device, they could distinguish between swallowing tasks of various samples, including saliva and water, and non-swallowing tasks, such as singing, chewing, speech, and isometric tongue push-up movements.

Minagi et al. (41) measured tongue pressure during swallowing in patients with PD using an ultra-thin sensor sheet with five pressure sensors affixed to the hard palate. The maximal tongue pressure at the measurement point was significantly lower in patients with PD than in healthy controls. Maximal tongue pressure was lower in patients with PD with dysphagia than in patients with PD without dysphagia. Loss of tongue pressure production in the anterior region of the hard palate was strongly associated with dysphagia in the oral and pharyngeal phases. They reported that abnormal tongue pressure production patterns, including partial or complete loss of tongue pressure, were observed at a higher rate in patients with PD with dysphagia than in patients with PD without dysphagia. They concluded that measuring tongue pressure during swallowing can detect not only the changes associated with overt dysphagia, but also the decreased tongue movement present in subclinical dysphagia.

**Figure 2:**
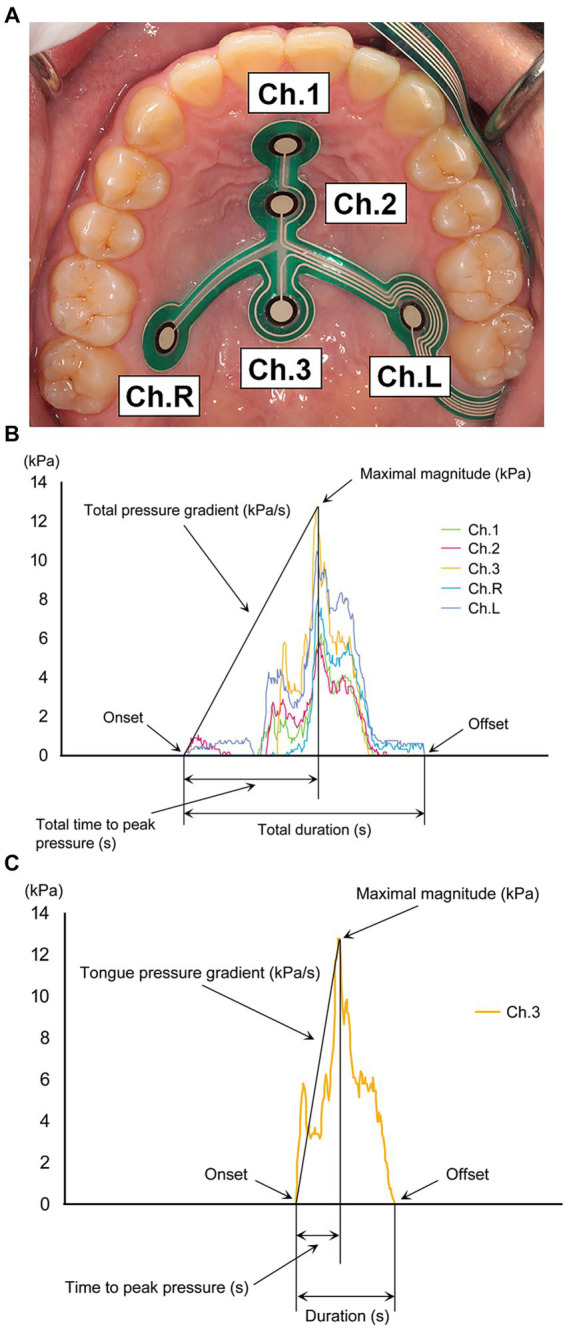
**(A)** Tongue pressure sensor sheet on the hard palate with five pressure-sensing parts. Ch.1 = anterior-median part; Ch.2 = mid-median part; Ch.3 = posterior-median part; Ch.R = right circumferential part; Ch.L = left circumferential part. **(B)** Tongue pressure waveforms of all channels recorded during swallowing. **(C)** Items for measuring tongue pressure during swallowing in a single channel.

Fukuoka et al. (42) used the same sensor sheet as Minagi et al. (41) to examine the characteristics of tongue movement in patients with PD (Figure 2). They compared tongue pressure during swallowing (maximal magnitude, duration, time-to-peak pressure, and pressure gradient) in dysphagia and non-dysphagia groups based on the findings of a videofluoroscopic swallowing study. No difference in maximal pressure was found between the two groups, but the duration and time-to-peak pressure were prolonged and the pressure gradient was decreased. These parameters may indicate temporal abnormalities in tongue movement in patients with PD. They concluded that measuring tongue pressure during swallowing using a tongue pressure sensor sheet can detect abnormal tongue movements in patients with PD, and is therefore useful in the diagnosis and treatment of dysphagia.

Other devices that may be able to measure tongue pressure during swallowing include the KayPentax 3-bulb array or the Madison Oral Strengthening Therapeutic device, but no data were found from studies with patients with PD (12, 43).

## 5 Discussion

Most patients with PD have disorders related to tongue pressure, and tongue strength and endurance, as well as tongue pressure during swallowing, are known to be related to swallowing efficiency and safety (11, 29–32, 34, 41, 42). Tongue strength and endurance are reduced by physiologic factors other than aging and worsen as PD progresses (31, 34). Decreased tongue strength and endurance in PD may be related to the cardinal features of basal ganglia dysfunction, such as bradykinesia and hypokinesia (32). Muscle strength, especially in the anterior part of the tongue, is retained in the early stages of PD (31), but declines in the more severe stages and may be a leading indicator with respect to sensitivity to disease progression.

Tongue pressure is associated with subjective symptoms of swallowing and eating-related quality of life. Patients with PD with impaired tongue pressure have lower SWAL-QOL scores because of the effects on items such as eating duration, food selection, symptom frequency, and eating desire (32, 36). Because these subjective symptoms can be attributed to dysphagia, tongue pressure should be evaluated for the purpose of detecting dysphagia in patients with PD.

Data on muscle strength in the posterior part of the tongue have not been fully accumulated, and there is room for further study of its diagnostic significance. In addition, there are few reports of studies on tongue endurance compared with tongue strength. Although there have been reports suggesting an association between decreased anterior tongue endurance and delayed laryngeal vestibular closure, the relationship between tongue endurance and swallowing function remains unclear (33). Investigating the effects of reduced muscle strength and endurance on swallowing efficiency and safety for the anterior and posterior portions of the tongue, respectively, could clarify whether tongue pressure is a clinical marker for the presence or absence of dysphagia and the pathophysiology of PD. Because tongue pressure measuring devices can provide visual feedback in the form of numerical values and waveforms, they are expected to be used in rehabilitation therapy, such as for strengthening the tongue muscles. Several studies in older adults and stroke patients with dysphagia have reported increased tongue strength, reduced pharyngeal residuals, decreased aspiration, and improved quality of life related to swallowing function following tongue strength training (14–16, 44–46). In the future, it will be necessary to study the effects of improved tongue strength and endurance through rehabilitation on improved swallowing function, subjective symptoms, and quality of life.

Measuring tongue pressure during swallowing is a method of assessing tongue–palate contact pressure during the swallowing of food and drinks, and evaluates tongue dynamics differently from measurements of tongue muscle strength and endurance. Tongue pressure during swallowing has been measured by various devices in patients with PD, all of which show a decrease compared with healthy older adults of the same age. It is important to note that the bulb-type measuring device only measures the tongue–palate contact pressure at one location during swallowing. In addition, because the patient swallows with the bulb in place, there is added resistance to tongue movement, which may induce unusual swallowing dynamics. The sensor sheet and experimental palatal plate are shaped to fit the palate, which allows simultaneous measurement of multiple tongue–palate contact sites.

The measurement of tongue pressure during swallowing using a sensor sheet has revealed abnormal tongue movement in patients with PD. Compared with healthy older adults and patients with PD without dysphagia, patients with PD with dysphagia have partial or complete deficits in tongue pressure during swallowing and prolonged tongue–palate contact time and time-to-peak pressure (41, 42). Patients with PD tend to have abnormal tongue pressure during swallowing, which is important for propelling a food bolus from the oral cavity to the pharynx, as the efficiency of swallowing is reduced. These features identified in the tongue pressure waveform are consistent with the dysphagia findings in videofluoroscopic swallowing studies, as well as in the poor and uncoordinated food feeding movements in the oral phase of swallowing in patients with PD (7, 9, 10, 47).

Measuring tongue pressure during swallowing may provide an assessment of how the normal pattern is disrupted compared with normal subjects based on changes in the order of tongue pressure onset, duration, and maximal magnitude. If the changes in swallowing-related organ movements can be quantitatively assessed with high sensitivity, it may be possible to identify and effectively respond to dysphagia in patients with PD from the early stage.

A remaining challenge is that most devices that measure tongue pressure during swallowing were developed for research purposes or are not commercially available. Direct comparisons or conversions of pressure measurements between these devices has not yet been established. In the future, it will be necessary to develop commercial instruments that can be shared by researchers and to integrate existing data for further analysis.

Tongue pressure measurement is an excellent assessment of tongue strength and tongue-palate contact during swallowing. However, it is challenging to measure involuntary movements, such as resting tremor and dyskinesia, which are characteristic of PD patients. A comprehensive evaluation of swallowing function requires a multifaceted examination that extends beyond tongue pressure alone. Integrating tongue pressure measurement into clinical observation and imaging evaluations may enable a more detailed analysis of swallowing function in PD patients.

## Author contributions

TF: Conceptualization, Funding acquisition, Investigation, Methodology, Writing – original draft, Writing – review & editing. KH: Conceptualization, Investigation, Methodology, Writing – original draft. TO: Conceptualization, Supervision, Writing – review & editing.
